# Diacerein versus non-steroidal anti-inflammatory drugs in the treatment of knee osteoarthritis: a meta-analysis

**DOI:** 10.1186/s13018-023-03786-6

**Published:** 2023-04-18

**Authors:** Fan Zeng, Kang Wang, Hang Duan, Xiao-tong Xu, Gao-yan Kuang, Min Lu

**Affiliations:** 1 The First Hospital of Hunan University of Chinese Medicine, Changsha, 410007 Hunan China; 2 Hunan University of Chinese Medicine, Changsha, 410208 Hunan China; 3 Huaihua Hospital of Traditional Chinese Medicine, Huaihua, 418000 Hunan China

**Keywords:** Knee osteoarthritis, Diacerein, Systematic review, Meta-analysis

## Abstract

**Background:**

Knee osteoarthritis (KOA) is a common musculoskeletal condition affecting millions of people worldwide and posing a significant challenge to clinicians and researchers. Emerging evidence suggests that the multifaceted symptomatology of KOA may be alleviated by diacerein. With this in mind, we conducted a systematic review and meta-analysis to evaluate the efficacy and safety of diacerein in patients with KOA.

**Methods:**

We systematically searched Embase, PubMed, Cochrane Library, Web of Science, Chinese Biomedical Literature Database (CBM), Wanfang Database (WanFang), China National Knowledge Infrastructure (CNKI), and China Science and Technology Journal Database (VIP) from their inception to August 2022 for randomized controlled trials (RCTs) of diacerein intervention on patients with KOA. Two reviewers independently performed the selection of eligible studies and the extraction of relevant data. The meta-analysis was performed using RevMan 5.4 and R 4.1.3 software tools. Depending on the type of outcome indicator selected, summary measures were expressed as mean differences (MD), standardized mean differences (SMD), or odds ratio (OR) with 95% confidence intervals (CI).

**Results:**

Twelve RCTs with 1732 patients were included. The results revealed that diacerein had comparable efficacy to non-steroidal anti-inflammatory drugs (NSAIDs) in reducing pain indicators such as Western Ontario and McMaster Universities Osteoarthritis Index (WOMAC) (SMD = 0.09, 95% CI [−0.10, 0.28], P = 0.34) and visual analogue scale (VAS) (SMD = −0.19, 95% CI [−0.65, 0.27], P = 0.42). However, diacerein outperformed NSAIDs in terms of global efficacy assessment by both patients and investigators (patients: 1.97, 95% CI [1.18, 3.29], P = 0.01; investigator: 2.18, 95% CI [0.99, 4.81], P = 0.05) at the end of treatment and sustained effectiveness in reducing WOMAC score and VAS score at four weeks after treatment. Moreover, there was no significant difference in adverse events incidence between the diacerein and NSAID groups. However, the GRADE evaluation indicated that the majority of the evidence quality was low.

**Conclusions:**

The results of this study suggest that diacerein could potentially be considered as a pharmacological agent with significant efficacy for the treatment of patients suffering from KOA, offering a potential alternative treatment strategy for those patients contraindicated to NSAIDs. However, further high-quality studies with longer follow-up are needed to make more informed decisions about its efficacy in the treatment of KOA.

**Supplementary Information:**

The online version contains supplementary material available at 10.1186/s13018-023-03786-6.

## Introduction

Knee osteoarthritis (KOA) is a common joint disease characterized by gradual cartilage degeneration leading to joint degeneration, pain, stiffness and limited range of motion in the knee [1]. With the growing aging population, the incidence of KOA is increasing annually, making it one of the leading causes of mobility loss in the elderly [2]. Recently, a study was conducted to assess the age-standardized prevalence of osteoarthritis in various countries in 2017. The study found that the prevalence ranged from 2090.3 to 6128.1 cases per 100,000 people, with the highest rates in the United States, American Samoa, and Kuwait. The escalating burden of osteoarthritis has a significant impact on healthcare systems and the economy, with medical costs associated with the disease accounting for 1–2.5% of gross domestic product (GDP) in high-income North American countries [3]. Given the lack of curative treatments for osteoarthritis, the current strategy is centered on alleviating pain and minimizing functional limitations. Although non-steroidal anti-inflammatory drugs (NSAIDs) are commonly used to manage osteoarthritis symptoms, their long-term use has been associated with an increased risk of gastrointestinal damage, cardiovascular events and recurrence of joint symptoms after discontinuation [4]. Therefore, there is an urgent need to find a safe and effective treatment option that can improve the symptoms of KOA.

Diacerein, an anthraquinone derivative extracted from rhubarb, has been used in recent years as a clinical treatment for various osteoarthritis conditions. Some studies suggest that diacerein has anti-catabolic properties acting on synovial membranes and cartilage [5–7]. However, despite the anti-inflammatory and anti-catabolic properties of diacerein on these tissues, several studies have questioned its efficacy in alleviating pain and improving functionality in patients with KOA, Specifically, these studies found no significant difference between the efficacy of diacerein and placebo in the management of KOA pain symptoms [8]. Hence, the efficacy of diacerein in the treatment of KOA remains controversial.

Therefore, this study conducted a meta-analysis by including randomized controlled trials of diacerein in the treatment of KOA, aiming to evaluate the effectiveness and safety of diacerein in the treatment of KOA, and to provide reference for clinical application.

## Material and methods

This study was conducted by the order of the PRISMA [9] and registered on PROSPERO (no. CRD42022365623).

## Literature search strategy

In this study, eight databases were searched by computer, including Embase, PubMed, Cochrane Library, Web of Science, Chinese Biomedical Literature Database (CBM), Wanfang Database (WanFang), China National Knowledge Infrastructure (CNKI), Chinese Science and Technology Periodical Database (VIP), and the search period is from their inception to August 2022. Our search strategy includes keywords such as "Osteoarthritis, Knee", "Knee Osteoarthritis", "diacerein" and "diacerhein", both in English and Chinese. The search terms for all databases are documented in Additional file 1.

## Inclusion/exclusion criteria

A literature must meet all of the following criteria to be considered: (1) Patients included in the study must meet the diagnostic criteria for osteoarthritis (The American College of Rheumatology (ACR) or Chinese Orthopedic Association-Guideline for diagnosis and treatment of osteoarthritis (COA); (2) Randomized controlled trials (RCTs), limited to Chinese and English literature; (3) The experimental group was given diacerein and the control group was given NSAIDs for at least 12 weeks. (4) Outcome indicators include at least one of the following: WOMAC, VAS, Global efficacy judgements by the patients and the investigator, and adverse effects.

The exclusion criteria were as follows: (1) Reviews and trials published only as abstracts. (2) Repeated publication of only one article retained. (3) The duration of the drug intervention was not the same in the experimental and control groups. (4) Full text or experimental data were not available. (5) Case reports, conference abstracts, commentaries, study protocols, and animal experiments.

## Study selection and data extraction

The literature was screened according to inclusion criteria by 2 investigators, and disagreements were resolved by open discussion if any. Among the information extracted from the literature were first author, year of publication, sample size, age, diagnostic criteria, interventions, main outcomes, and adverse effects. The first author of the literature was contacted if any information was missing.

## Quality assessment

The quality of the included literature was assessed by applying the revised Cochrane Risk of Bias tool (RoB 2.0) [10]. The RoB table comprised five domains, namely, bias arising from the randomization process, bias in the measurement of the outcome, bias due to deviations from intended interventions, bias from missing data, and bias in the selection of the reported results. Each trial was systematically assessed for the risk of bias and categorized as either high risk, low risk, or some concerns based on the answers to signaling questions. Two reviewers independently assessed the quality of each literature and disagreements were resolved by open discussion.

## Statistical analysis

This study used RevMan 5.4 and R (version 4.1.3) for data analysis. For continuous data (e.g., WOMAC and VAS), outcome indicators were expressed as mean differences (MD), or standardized mean differences (SMD) if the study used different unit measurement scales. For dichotomous data (e.g., Global efficacy judgements by the patients and the investigator, and adverse effects), outcome indicators were expressed as odds ratio (OR) and 95% confidence intervals (CIs). All meta-analyses used a random effects method, and the results of the fixed-effects model are presented in Additional file 2. I^2^ and P tests were used to evaluate the heterogeneity among the studies. Publication bias was detected by visual funnel plots and Peters test [11].

## Quality of evidence

The general quality of the outcomes was assessed utilizing the Grades of Recommendation, Assessment, Development, and Evaluation (GRADE) system [12]. The GRADE system took into account various factors including study design, imprecision, risk of bias, inconsistency, indirectness, and other considerations. The quality of evidence was finally categorized into four levels: high, moderate, low, or very low.

## Results

### Search results

**Fig. 1:**
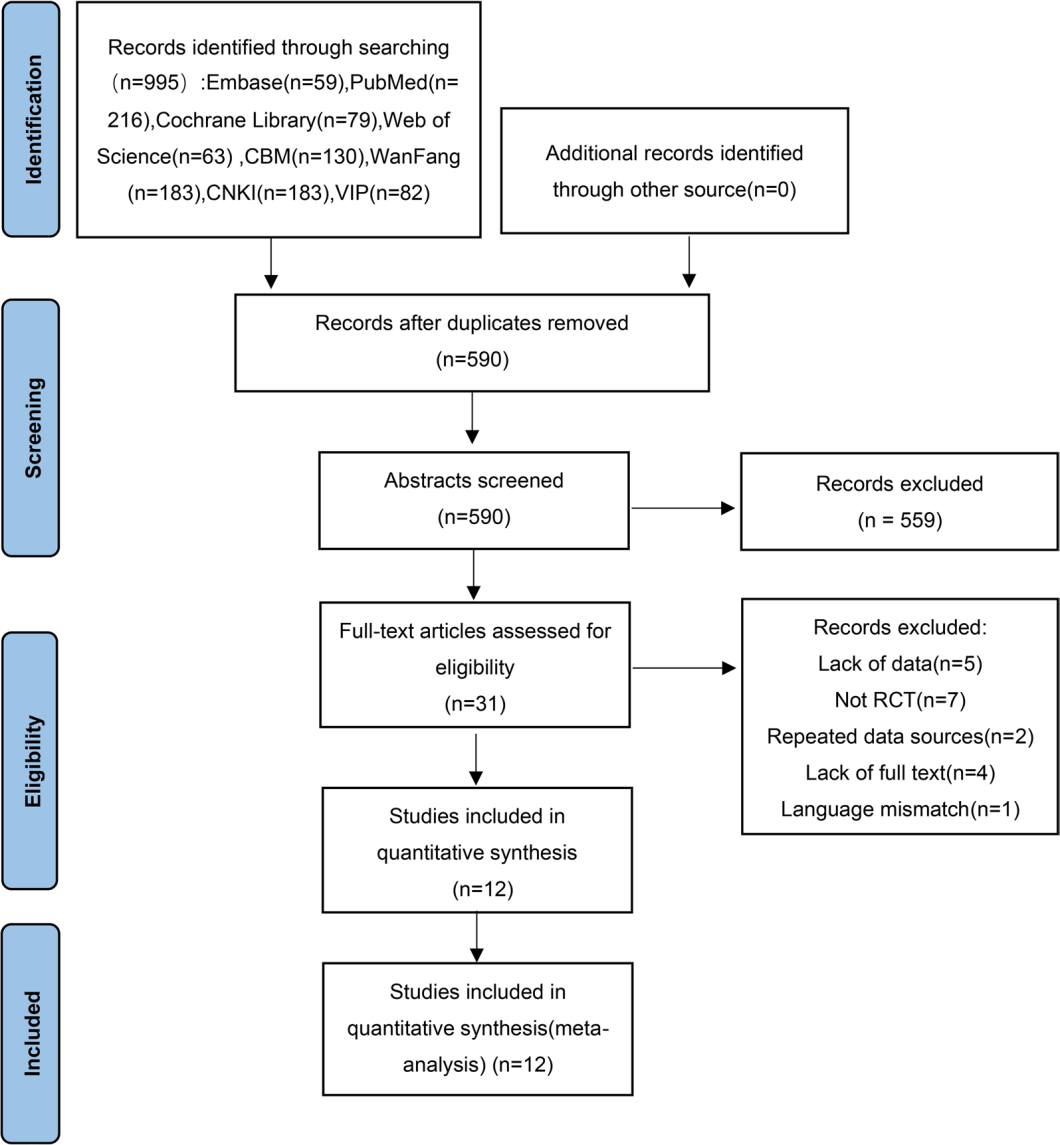
Flow chart for identifying eligible studies

The database was searched according to the developed search strategy and 995 potentially eligible studies were obtained. 590 publications were initially screened by removing duplicate studies. After browsing the abstract and reading the full text, 12 literatures that met the inclusion and exclusion criteria were finally included. The literature screening process is shown in Fig. 1.

## Characteristics of included studies

Twelve randomized controlled trials (RCTs) with a total of 1732 participants were included in this study, where 861 cases received diacerein as the experimental treatment and 871 cases received NSAIDs as the control treatment. The treatment duration for both groups was at least 12 weeks. The basic characteristics of the included studies are summarized in Table 1.

**Table 1:** Characteristics of included studies

Trail	Sample size (T/C)	Age (y), mean ± SD or median (range)		Diagnostic criteria	T	C	Duration	Main outcomes
		T	C					
Li et al. [13]	30/30	61.0 ± 8.14	60.97 ± 7.49	COA	diacerein	diclofenacSodium	12 weeks	①②③
Chen et al. [14]	70/70	55.99 ± 10.47	56.77 ± 10.60	COA	diacerein	celecoxib	12 weeks	①②
Wang [15]	37/38	58.2 ± 10.7		ACR	diacerein	diclofenacSodium	12 weeks	③
Chen et al. [16]	32/30	63(45–75)		ACR	diacerein	Loxoprofen	12 weeks	③
Chen [17]	18/18	56.57 ± 5.51		COA	Diacerein	celecoxib	12 weeks	①③
Ma [18]	37/38	55.3 ± 6.9	54.6 ± 7.1	ACR	diacerein	meloxicam	12 weeks	①②③
Zhang et al. [19]	21/21	43–73	46–72	ACR	diacerein	diclofenacSodium	12 weeks	①②③④
Ye and Xue [20]	180/180	40–65		ACR	diacerein	piroxicam	16 weeks	①②③④
Tian et al. [21]	54/54	NR		ACR	diacerein	diclofenacSodium	12 weeks	①②③
Pelletier et al. [22]	187/193	63.7 ± 6.3	64.4 ± 7.0	ACR	diacerein	Celecoxib	24 weeks	①②③
Zheng et al. [23]	111/112	58.22 ± 8.42	59.49 ± 8.56	ACR	diacerein	diclofenacSodium	12 weeks	①②③④
Louthrenoo et al. [24]	86/85	54 ± 6.2	54 ± 7.0	ACR	diacerein	Piroxicam	16 weeks	①③④

T: Trial Group;C: Control Group; VAS: visual analogue scale; WOMAC: Western Ontario and McMaster Universities Osteoarthritis Index; NR: not reported; ①WOMAC②VAS ③adverse reactions ④Global efficacy judgements by the patients and the investigator

## Quality assessment

**Fig. 2:**
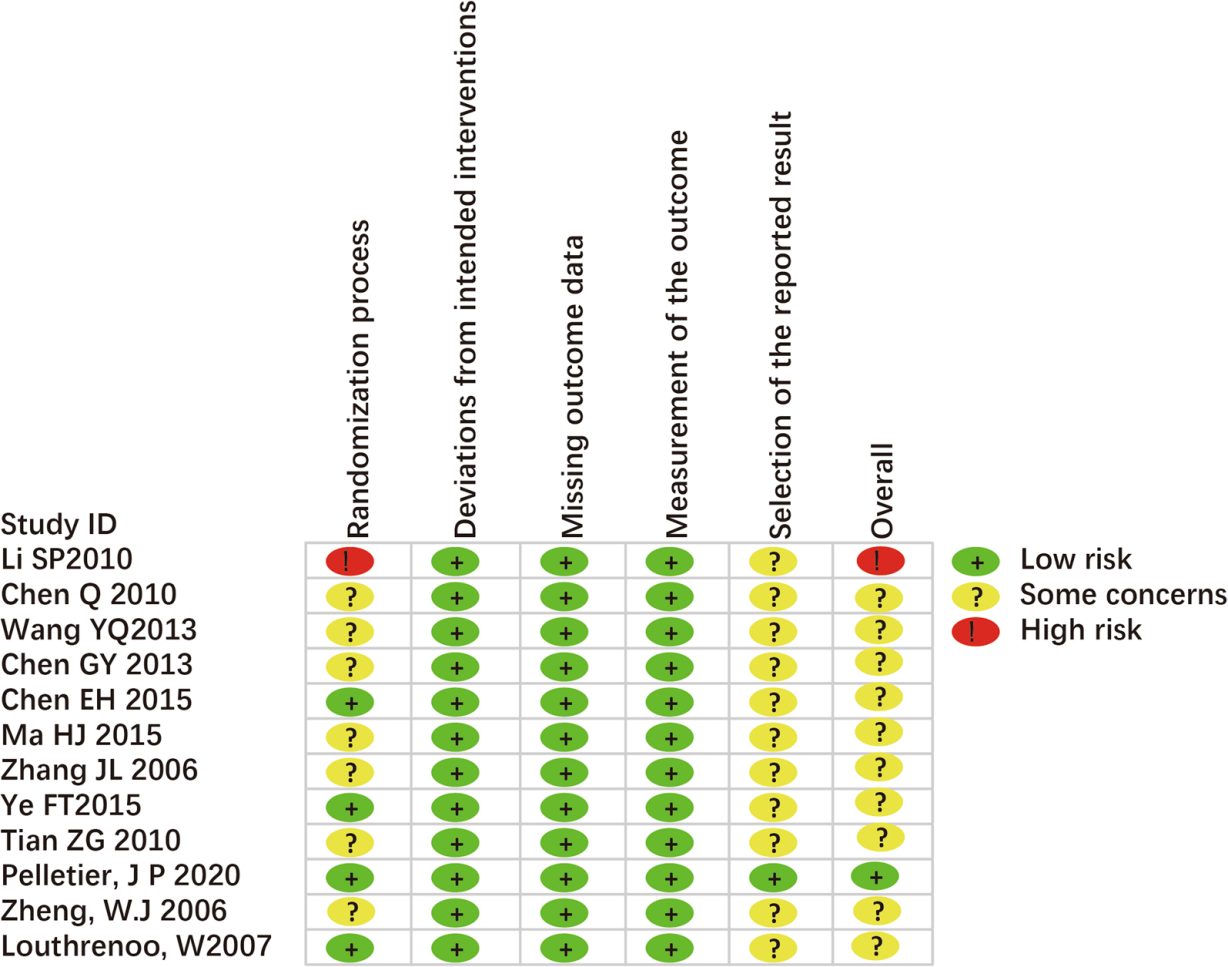
Risk of bias of individual study

**Fig. 3:**
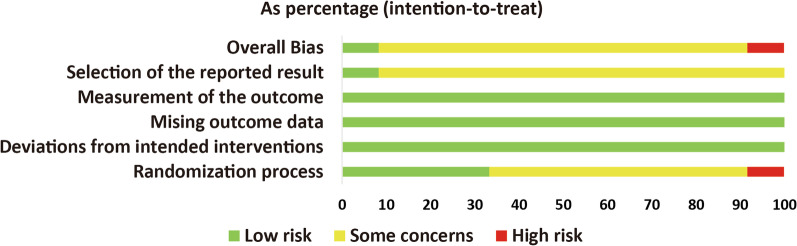
overall risk of bias

Of the 12 RCTs included in this study, five [13, 17, 20, 22, 24] described the methods of randomization used, which included the visit order, random number table method, and computerized random sequence generation method. However, one [13] of the studies used a "visit order" randomization strategy, which was associated with a high risk of bias in the process of randomization. The remaining seven studies were rated as having "some concerns" due to the lack of clear description of their randomization method. All RCTs were rated as low risk in the section of deviations from the intended interventions and outcome measures. Only one trial [22] provided a registration number of pre-registered protocol, and the “selection of reported results” was rated as low risk. The risk of bias assessment for the included RCTs is shown in Figs. 2 and 3.

## Meta-analysis results

### WOMAC score

**Fig. 4:**
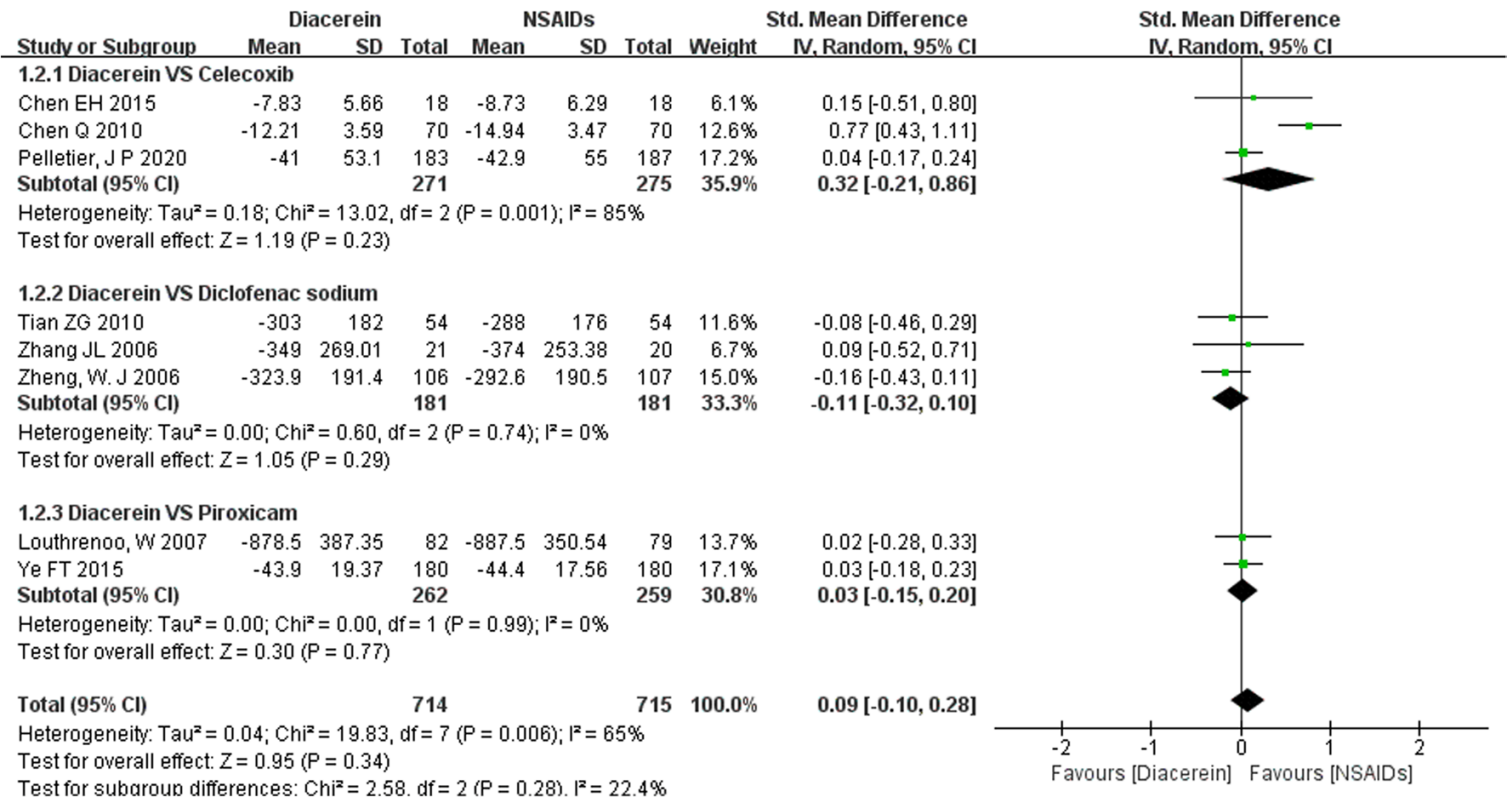
Forest plot of WOMAC

10 studies reported WOMAC [25] score. However, two [13, 18] of them used a non-standard WOMAC scale, which made it impossible to combine their results. Therefore, only eight studies [14, 17, 19–24] were included in the meta-analysis.. The pooled results showed that diacerein and NSAIDs had similar efficacy in improving the WOMAC score (SMD = 0.09, 95% CI [−0.10, 0.28], P = 0.34). The reliability of the results was confirmed by sensitivity analyses, which showed that no study significantly reversed the meta-analysis results. Furthermore, the subgroup analysis showed that diacerein had similar efficacy to celecoxib (SMD = 0.32, 95% CI [−0.21, 0.86], P = 0.23), diclofenac sodium (SMD = −0.11, 95% CI [−0.32, 0.10], P = 0.29), or piroxicam (SMD = 0.03, 95% CI [−0.15, 0.20], P = 0.77) in improving WOMAC score (Fig. 4).

**Fig. 5:**
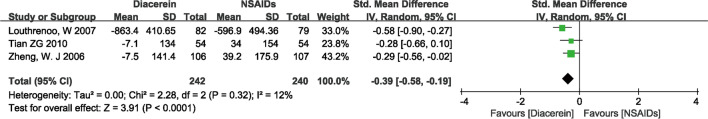
Forest plot of WOMAC score at 4-week follow-up after treatment

Three studies [21, 23, 24] reported the WOMAC score of patients at 4-week follow-up after treatment. The pooled results revealed a significant statistical difference in WOMAC score between the experimental and control groups (SMD = −0.39, 95% CI [−0.58, −0.19], P < 0.0001), suggesting that diacerein was more effective in improving WOMAC score than NSAIDs at 4 weeks after drug discontinuation (Fig. 5).

### VAS score

**Fig. 6:**
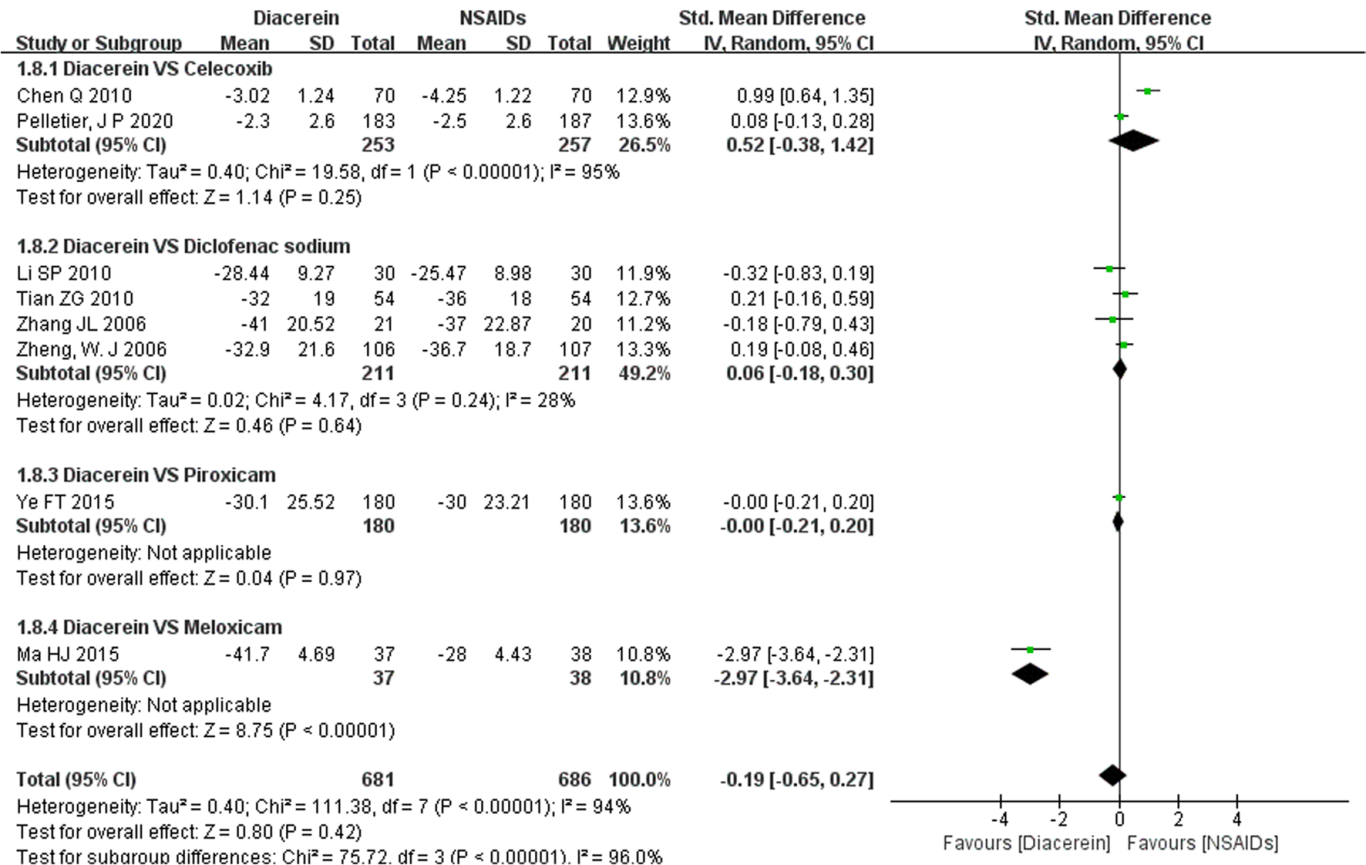
Forest plot of VAS Score

Eight studies [13, 14, 18–23] reported VAS [26] score. The VAS score between the experimental group and control groups was not statistically significant (SMD = −0.19, 95% CI [−0.65, 0.27], P = 0.42), suggesting that diacerein and NSAIDs had comparable effects in improving VAS score. A sensitivity analysis was performed due to the high heterogeneity among studies, and the results of the meta-analysis were not significantly reversed when studies were excluded one by one, indicating that the above results were reliable. Subgroup analysis revealed that diacerein was more effective in improving VAS scores compared to meloxicam (SMD = −2.97, 95% CI [−3.64, −2.31], P < 0.00001), but it was not superior to celecoxib (SMD = 0.52, 95% CI [−0.38, 1.42], P = 0.25), diclofenac sodium (SMD = 0.06, 95% CI [−0.18, 0.30], P = 0.64), or piroxicam (SMD = −0.00, 95% CI [−0.21, 0.20], P = 0.97) (Fig. 6).

**Fig. 7:**
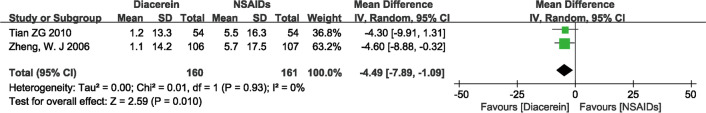
Forest plot of VAS score at 4-week follow-up after treatment

Two studies [21, 23] reported patients' VAS scores at 4 weeks of follow-up after the end of treatment.. The VAS score between the experimental group and control group was statistically significant (MD = −4.49, 95% CI [−7.89, −1.09], P = 0.01), suggesting that diacerein was more effective in improving VAS score than NSAIDs at 4 weeks after drug discontinuation, as shown in Fig. 7.

### Global efficacy judgements by the patients and the investigator

**Fig. 8:**
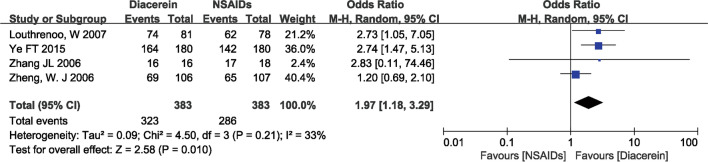
Forest plot of global efficacy judgements by the patients

**Fig. 9:**
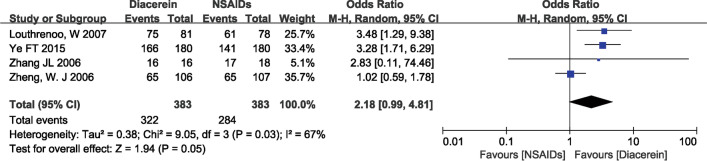
Forest plot of global efficacy judgements by the investigator

The overall effectiveness of a treatment is often assessed by both the patient and the investigator using a four-point rating scale (‘‘How successful do you think the treatment has been so far?” Response options range from “ineffective” to “slightly effective,” “moderately effective,” and “very effective.”) [24]. In four studies [19, 20, 23, 24], investigators' and patients' judgments of global efficacy were reported. The pooled results showed that diacerein was better than NSAIDs in terms of global efficacy judgments by patients and investigators (patients: 1.97, 95% CI [1.18, 3.29], P = 0.01; investigator: 2.18, 95% CI [0.99, 4.81], P = 0.05), as shown in Fig. 8 and 9.

### Safety

**Fig. 10:**
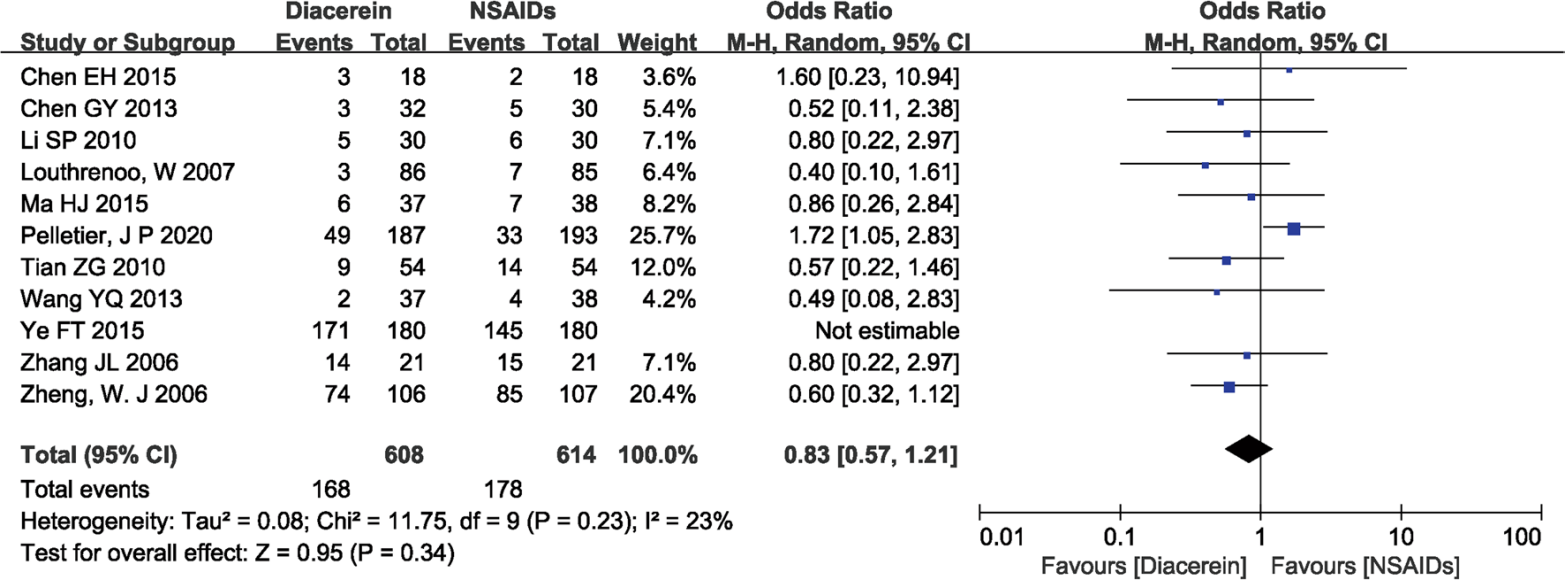
Forest plot of adverse effects

Adverse events were reported in 11 studies [13, 15–24], with the gastrointestinal system being the most affected, showing symptoms such as diarrhea, nausea, and abdominal pain. The pooled results showed that the safety of diacerein and NSAIDs was comparable (OR = 0.83, 95% CI [0.57, 1.21], P = 0.34), as shown in Fig. 10.

### Publication bias

**Fig. 11:**
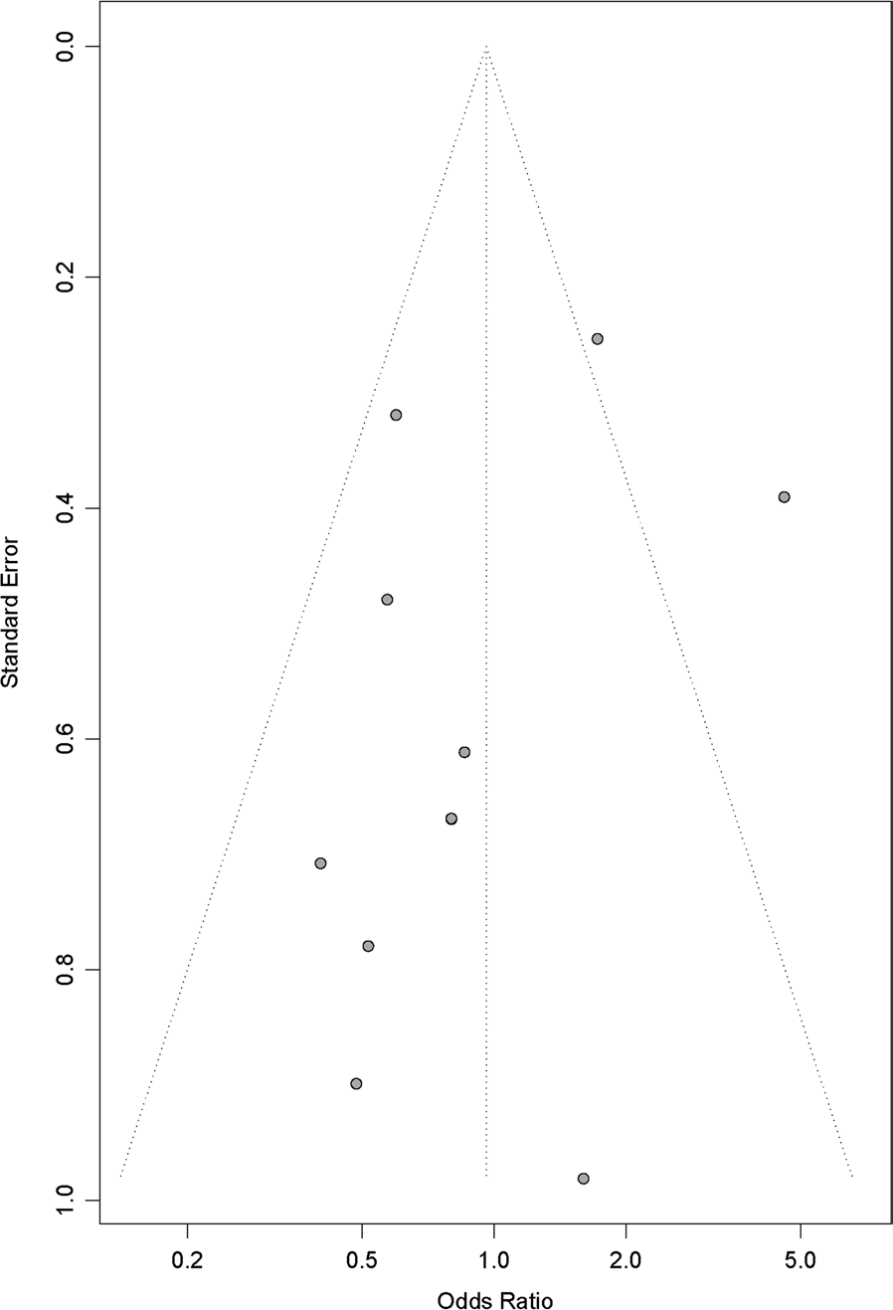
Publication-bias chart

With more than 10 studies reporting adverse events, we assessed publication bias by funnel plot and Peters test. A visual inspection of the funnel plot indicated that publication bias may have occurred (Fig. 11). Statistical significance was not determined by the Peters test (P = 0.1967), suggesting that publication bias was not evident.

### Quality assessment

The quality of evidence was evaluated using the GRADE pro tool. And the results are summarized in Table 2.

**Table 2:** The quality of evidence

Outcomes	Illustrative comparative risks* (95% CI)		Relative effect (95% CI)	No. of Participants (studies)	Quality of the evidence (GRADE)	Comments
	Assumed risk	Corresponding risk				
	NSAIDs	Diacerein				
WOMAC		The mean WOMAC in the intervention groups was 0.09 standard deviations higher (0.1 lower to 0.28 higher)		1429 (8 studies)	$\oplus \oplus$OO low^1,2,3^	SMD 0.09 (−0.1 to 0.28)
VAS		The mean vas in the intervention groups was 0.19 standard deviations lower (0.65 lower to 0.27 higher)		1367 (8 studies)	$\oplus \oplus$OO low^1,2,3^	SMD −0.19 (−0.65 to 0.27)
Global efficacy judgements by the patients	Study population		OR 1.97 (1.18 to 3.29)	766 (4 studies)	$\oplus \oplus$OO low^3,4,5^	
	747 per 1000	853 per 1000 (777 to 907)				
	Moderate					
	792 per 1000	882 per 1000 (818 to 926)				
Global efficacy judgements by the investigator	Study population		OR 2.18 (0.99 to 4.81)	766 (4 studies)	$\oplus \oplus$OO low^2,3,4^	
	742 per 1000	862 per 1000 (740 to 932)				
	Moderate					
	783 per 1000	887 per 1000 (781 to 946)				
Safety	Study population		OR 0.83 (0.57 to 1.21)	1222 (10 studies)	$\oplus$OOO very low^1,3,5^	
	290 per 1000	253 per 1000 (189 to 331)				
	Moderate					
	184 per 1000	158 per 1000 (114 to 214)				

*CI* Confidence interval; *OR* Odds ratio

GRADE Working Group grades of evidence

High quality: Further research is very unlikely to change our confidence in the estimate of effect

Moderate quality: Further research is likely to have an important impact on our confidence in the estimate of effect and may change the estimate

Low quality: Further research is very likely to have an important impact on our confidence in the estimate of effect and is likely to change the estimate

Very low quality: We are very uncertain about the estimate

^1^High risk of bias due to the lack of blinding

^2^High heterogeneity

^3^Wide CI

^4^Small sample size

^5^Moderate heterogeneity

## Discussion

In this study, the efficacy of diacerein in the treatment of KOA was systematically evaluated in terms of WOMAC score, VAS score, and global efficacy judgements by the patients and the investigator. Based on the results of the above meta-analysis, diacerein demonstrated good efficacy in improving WOMAC score, and VAS score, which was comparable to NSAIDs. Surprisingly, although there were no significant differences between diacerein and NSAIDs in terms of WOMAC and VAS scores during the treatment period, both patients and investigators perceived diacerein to be significantly more effective than NSAIDs in terms of overall efficacy. Additionally, our study found that diacerein was superior to NSAIDs in improving WOMAC score and VAS score during the follow-up period after the end of treatment, suggesting that it has a stronger follow-up effect. Moreover, compared with several past meta-analysis [27–29], our study developed stricter inclusion and exclusion criteria; in the current studies, the drugs in the control group were limited to NSAIDs, and the treatment population was limited to KOA patients. The treatment course of both the experimental group and the control group was more than 12 weeks, which effectively reduced the heterogeneity between studies and made the results more referential to clinicians.

KOA is primarily characterized by cartilage degeneration, with pro-inflammatory cytokines being a key factor in the development of the condition [30]. Elevated levels of pro-inflammatory factors in the joints have been identified as a facilitator of cartilage destruction [31]. Interleukin-1 (IL-1) has been detected in some joint tissues of KOA patients and has been shown to interfere with chondrocyte catabolism and anabolism, resulting in accelerated cartilage degradation metabolism and decreased cartilage anabolism. IL-1 also induces inflammation in synovial cells [32, 33]. Kobayashi et al. [34] showed that targeting the IL-1 receptor with IL-1β inhibitors inhibited the effect of IL-1β and reduced cartilage matrix degradation, thus promoting cartilage repair. Blom et al. [35] demonstrated that the number of macrophages was positively correlated with the formation of joint bone redundancy, and IL-1β could recruit other pro-inflammatory factors and chemokines, which increased macrophage infiltration, promoted local bone redundancy formation, and accelerated joint pathological damage. Moreover, Attur et al. [36] showed that OA patients with IL-1 overexpression had a higher number of affected joints, higher pain scores, and an increased risk of progression on imaging. In conclusion, IL-1 is closely associated with pain and disease progression in KOA patients.

Diacerein is an anthraquinone derivative, and its primary mechanism of action is to inhibit the IL-1 system and its associated downstream signaling pathways. Research by Moldovan et al. [37] has shown that diacerein can reduce the activation of IL-1β by decreasing the production of IL-1 converting enzyme. It also possesses anti-inflammatory properties by decreasing IL-1 receptor levels in chondrocytes and increasing the production of IL-11 receptor antagonist, which ultimately leads to lower IL-1 levels in the synovial fluid of knee osteoarthritis patients. Additionally, diacerein inhibits the MAPK pathway activated by IL-1 and the binding of transcription factors NF-kappaB and AP-1. These factors are crucial in the expression of several pro-inflammatory genes in chondrocytes [38]. Numerous studies have also demonstrated the cartilage protective effects of diacerein. Boileau et al. [39] demonstrated that diacerein effectively prevents cartilage degradation by reducing MMP-13 activity and osteoclast formation. In various animal models of OA, diacerein was found to be effective in reducing cartilage loss, ameliorating cartilage lesions, and delaying arthritis progression in meniscectomy-induced OA rat models compared to untreated controls [40, 41]. Thus, diacerein is a promising therapeutic agent for the treatment of KOA.

Eleven studies reported adverse events, and meta analysis showed that the incidence of adverse events with diacerein was comparable to that of NSAIDs, signifying a favorable safety profile. However, among the adverse events reported in various studies, it can be found that the incidence of upper gastrointestinal adverse events of NSAIDs is significantly higher than that of diacerein. For example, Louthreno et al. [24] reported that one NSAID-treated patient was hospitalized for gastrointestinal bleeding during treatment, whereas no serious adverse events were reported in the diacerein-treated patients. NSAIDs exert analgesic, anti-inflammatory and antipyretic effects by inhibiting prostaglandin synthesis, which may lead to various adverse effects such as gastrointestinal complications (perforation, ulceration, and bleeding) and an increased risk of cardiovascular events [42]. However, diacerein has a limited impact on prostaglandin synthesis and the upper gastrointestinal mucosa. Diarrhea is the primary adverse effect associated with diacerein and is generally mild and transient. This is a notable advantage over NSAIDs and supports diacerein as a viable alternative to NSAIDs in the treatment of OA, particularly in elderly patients and those at increased risk for gastrointestinal bleeding and cardiovascular disease.

### Limitations

This study has several limitations that need to be acknowledged. (1) The reliability of the conclusions may be affected by the small volume of included literature; (2) The outcome indicators of the included studies are mostly subjective, which may affect the accuracy of the results. (3) Methodology limitations existed in most of the included studies, such as unclear bias risks in random sequence generation and blinding. (4) No long-term follow-up studies have been conducted, and only one study included a 6-month study period. The long-term efficacy and safety of Diacerein remain to be determined. (5) The level of evidence for most outcomes was rated as "low" according to GRADE.

## Conclusion

In conclusion, the present study provides evidence that diacerein could potentially be considered as a pharmacological agent with significant efficacy for the treatment of patients suffering from KOA, offering a potential alternative treatment strategy for those patients contraindicated to NSAIDs. However, the conclusions of this study need to be further verified by large-sample, multi-center, and long-term follow-up clinical studies.

## Supplementary Information

**Additional file 1:** Search strategies.

**Additional file 2:** Results of fixed effects model.

## Data Availability

The data used to support the findings of this study are available from the corresponding author on reasonable request.

## Abbreviations

KOA: Knee osteoarthritis
CBM: Chinese Biomedical Literature Database
CNKI: China National Knowledge Infrastructure
VIP: Chinese Science and Technology Periodical Database
RCTs: Randomized controlled trials
NSAIDS: Non-steroidal anti-inflammatory drugs
WOMAC: Western Ontario and McMaster Universities Osteoarthritis Index
VAS: Visual analogue scale
MD: Mean differences
SMD: Standardized mean differences
OR: Odds ratio
CI: Confidence intervals
GDP: The Gross Domestic Product
PRISMA: Preferred Reporting Items for Systematic Reviews and Meta-Analysis
