# Supplementary material for: Diacerein versus non-steroidal anti-inflammatory drugs in the treatment of knee osteoarthritis: a meta-analysis
Source: J Orthop Surg Res. 2023 Apr 18;18:308. doi: 10.1186/s13018-023-03786-6 (PMC10114432; doi:10.1186/s13018-023-03786-6)
Supplement: Supplementary file 1 — Additional file 1: Search strategies. [file 13018_2023_3786_MOESM1_ESM.pdf]

Supplement 1. Search strategies

Embase:

1. 'knee osteoarthritis'/exp
2. 'knee osteoarthritides':ab,ti OR 'knee osteoarthritis':ab,ti OR 'osteoarthritis of knee':ab,ti OR 'osteoarthritis of the knee':ab,ti
3. #1 OR #2
4. 'diacerein'/exp
5. 'diacetylrhein':ab,ti OR 'diacerhein':ab,ti OR 'diacetyl-rhein':ab,ti OR '4,5-diacetoxyanthraquinone-2-carboxylic acid':ab,ti OR '1,8-diacetoxy-3-carboxyanthraquinone':ab,ti OR 'sf 277':ab,ti OR 'rhein diacetate':ab,ti
6. #4 OR #5
7. 'randomized controlled trial':ab,ti OR 'randomized':ab,ti OR 'placebo':ab,ti
8. #3 AND #6 AND #7

PUBMED:

1. ("Osteoarthritis, Knee"[Mesh]) OR ((((Knee Osteoarthritides[Title/Abstract]) OR (Knee Osteoarthritis[Title/Abstract])) OR (Osteoarthritis of Knee[Title/Abstract])) OR (Osteoarthritis of the Knee[Title/Abstract]))
2. ((((((diacetylrhein[Title/Abstract]) OR (diacerhein[Title/Abstract])) OR (diacetyl-rhein[Title/Abstract])) OR (4,5-Diacetoxyanthraquinone-2-carboxylic Acid[Title/Abstract])) OR (1,8-Diacetoxy-3-carboxyanthraquinone[Title/Abstract])) OR (SF 277[Title/Abstract])) OR (rhein diacetate[Title/Abstract])
3. randomized controlled trial[Publication Type] OR randomized[Title/Abstract] OR placebo[Title/Abstract]
4. #1 AND #2 AND #3

WEB OF SCIENCE:

1. TS=(Osteoarthritis, Knee OR Knee Osteoarthritides OR Knee Osteoarthritis OR Osteoarthritis of Knee OR Osteoarthritis of the Knee)
2. TS=(diacetylrhein OR diacerhein OR diacetyl-rhein OR 4,5-Diacetoxyanthraquinone-2-carboxylic Acid OR 1,8-Diacetoxy-3-carboxyanthraquinone OR SF 277 OR rhein diacetate OR diacerein)
3. TS=(randomized controlled trial OR randomized OR placebo)

#1 AND #2 AND #3

The Cochrane library:

#1 MeSH descriptor: [Osteoarthritis, Knee] explode all trees
#2 (Knee Osteoarthritides):ti,ab,kw OR (Knee Osteoarthritis):ti,ab,kw OR (Osteo

arthritis of Knee):ti,ab,kw OR (Osteoarthritis of the Knee):ti,ab,kw
#3 (diacetylrhein):ti,ab,kw OR (diacerhein):ti,ab,kw OR (diacetyl rhein):ti,ab,kw OR (4,5 Diacetoxyanthraquinone 2 carboxylic Acid):ti,ab,kw OR (1,8 Diacetoxy 3 carboxyanthraquinone):ti,ab,kw OR (SF 277):ti,ab,kw OR (rhein diacetate):ti,ab,kw OR (diacerein):ti,ab,kw
#4 #1 OR #2
#5 #4 AND #3

China National Knowledge Infrastructure (CNKI):

((((（主题=骨关节炎，膝 或者 题名=骨关节炎，膝 或者 v_subject=中英文扩展(骨关节炎，膝) 或者 title=中英文扩展(骨关节炎，膝)) 或者 (主题=膝骨关节炎 或者 题名=膝骨关节炎 或者 v_subject=中英文扩展(膝骨关节炎) 或者 title=中英文扩展(膝骨关节炎))) 或者 ((主题=膝骨性关节炎 或者 题名=膝骨性关节炎 或者 v_subject=中英文扩展(膝骨性关节炎) 或者 title=中英文扩展(膝骨性关节炎)) 或者 (主题=膝关节骨性关节炎 或者 题名=膝关节骨性关节炎 或者 v_subject=中英文扩展(膝关节骨性关节炎) 或者 title=中英文扩展(膝关节骨性关节炎)))) 或者 ((主题=膝关节退行性关节炎 或者 题名=膝关节退行性关节炎 或者 v_subject=中英文扩展(膝关节退行性关节炎) 或者 title=中英文扩展(膝关节退行性关节炎)) 或者 (主题=膝关节骨关节炎 或者 题名=膝关节骨关节炎 或者 v_subject=中英文扩展(膝关节骨关节炎) 或者 title=中英文扩展(膝关节骨关节炎)))) 或者 (主题=膝关节退行性病变 或者 题名=膝关节退行性病变 或者 v_subject=中英文扩展(膝关节退行性病变) 或者 title=中英文扩展(膝关节退行性病变))) 并且 ((摘要=双醋瑞因 或者 abstract_en=中英文扩展(双醋瑞因)) 或者 (摘要=安必丁 或者 abstract_en=中英文扩展(安必丁))) 并且 (((摘要=随机对照试验 或者 abstract_en=中英文扩展(随机对照试验)) 或者 (摘要=随机对照 或者 abstract_en=中英文扩展(随机对照))) 或者 (摘要=随机 或者 abstract_en=中英文扩展(随机)))

Wanfang Database(WanFang):

((主题:(骨关节炎，膝) or 主题:(膝骨关节炎) or 主题:(膝骨性关节炎) or 主题:(膝关节骨性关节炎) or 主题:(膝关节退行性关节炎) or 主题:(膝关节骨关节炎))and(主题:(双醋瑞因) or 主题:(安必丁)) and (主题:(随机对照试验) or 主题:(随机对照) or 主题:(随机))

Chinese Science and Technology Periodical Database (VIP):

(题名或关键词=骨关节炎，膝 OR 题名或关键词=膝骨关节炎) OR 题名或关键词=膝骨性关节) OR 题名或关键词=膝关节骨性关节炎) OR 题名或关键词=膝关

节退行性关节炎) OR 题名或关键词=膝关节骨关节炎) OR 题名或关键词=膝关节退行性病变) AND (题名或关键词=双醋瑞因 OR 题名或关键词=安必丁)) AND ((摘要=随机对照试验 OR 摘要=随机对照) OR 摘要=随机))

Chinese Biomedical Literature Database (CBM):

1) "骨关节炎，膝"[不加权:扩展]

2) ("膝骨关节炎"[常用字段:智能] OR "膝骨性关节炎"[常用字段:智能] OR "膝关节骨性关节炎"[常用字段:智能] OR "膝关节退行性关节炎"[常用字段:智能] OR "膝关节骨关节炎"[常用字段:智能] OR "膝关节退行性病变"[常用字段:智能])

3) ("双醋瑞因"[常用字段:智能] OR "安必丁"[常用字段:智能])

4) "随机对照试验"[不加权:扩展]

5) ("随机对照"[常用字段:智能] OR "随机"[常用字段:智能])

6) ((#1) OR (#2))

7) ((#4) OR (#5))

8) ((#7) AND (#6) AND (#3))
